# lncRNA LINC01315 promotes malignancy of triple-negative breast cancer and predicts poor outcomes by modulating microRNA-876-5p/GRK5

**DOI:** 10.1080/21655979.2022.2062536

**Published:** 2022-04-12

**Authors:** Yan Xiu, Shannan Cao, Ru Jiang, Yuming Zhou

**Affiliations:** Medical Clinical Laboratory, Yantai Affiliated Hospital of Binzhou Medical University, Yantai, Shandong, China

**Keywords:** Triple-negative breast cancer, lncRNA LINC01315, miR-876-5p, prognosis, disease development, tumor progression

## Abstract

Triple-negative breast cancer (TNBC) is a malignant tumor that threatens women’s health. Exploring novel development-associated biomarkers could help improve the survival rate of TNBC. This study evaluated the significance and mechanism of LINC01315 in TNBC progression aiming to identify a potential biomarker. There were 103 TNBC patients that provided clinical tissues in this study. The expression of LINC01315 was assessed by PCR and its association with clinical data was evaluated by statistical analyses. The *in vitro* cell experiments were conducted to estimate the biological effect of LINC01315 and its molecular mechanism. A significant upregulation of LINC01315 was observed in TNBC, which was associated with disease development and severity of patients. The upregulation of LINC01315 could be a symptom of the poor prognosis of patients. The knockdown of LINC01315 suppressed the main cellular processes of TNBC progression. Additionally, miR-876-5p was demonstrated to be a target of LINC01315 and regulate the expression of GRK5, through which LINC01315 modulated the progression of TNBC. Upregulated LINC01315 in TNBC indicated the malignant development and poor survival rate of patients. Inhibition of LINC01315 might be a potential therapeutic strategy of TNBC.

## GRAPHICAL ABSTRACT

**Figure:**
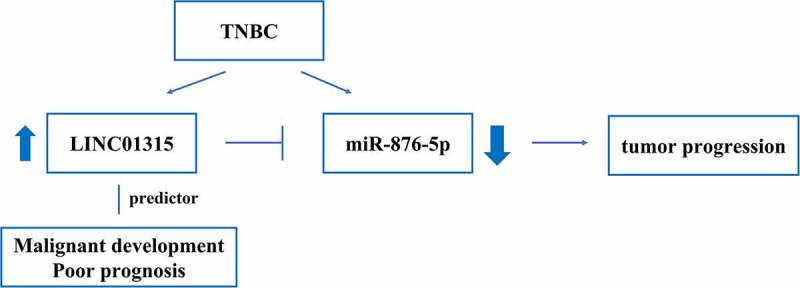

## Introduction

Among various subtypes of breast cancer, triple-negative breast cancer (TNBC) is one of the most malignant subclasses with high mortality and aggressiveness [1]. Although the clinical therapeutic strategy of TNBC has been ameliorated in the past decades, the clinical prognosis of TNBC patients was still unsatisfying [2]. With the lack of obvious symptoms in early detection, patients are always diagnosed at an advanced stage, which is the major factor responsible for the poor prognosis [3]. Identifying novel effective biomarkers could not only improve the survival of patients but also provide a potentially promising target for the therapy of TNBC.

Latter progression in molecular biology leaked out a large number of non-coding RNAs that play roles in the development of various cancers. Long non-coding RNAs (lncRNAs) are in a length of over 200 nucleotides and are short of the ability to code proteins [4]. Nowadays, increasing studies have been devoted to digging out key genes associated with the clinical outcomes of cancer patients [5,6]. Recent studies have reported several lncRNAs that could monitor tumor progression of TNBC and predict the overall survival of patients [7]. For example, abnormally expressed lncRNA LINC01087 could distinguish TNBC patients from luminal breast cancer patients and act as a predictive biomarker for the outcome and pharmacological interventions of patients [8]. LncRNA GATA3-AS1 showed a significant promoted effect on the progression and immune evasion of TNBC via regulating PD-L1 and GATA3 [9]. The dysregulation of LncRNA LINC01315 (LINC01315) has been observed in breast cancer, intimating its potential in indicating TNBC development and prognosis [10,11]. Even though LINC01315 has been illustrated to inhibit the development of oral squamous cell carcinoma [12], accelerate the aggressive phenotypes of papillary thyroid cancer cells [13], and regulate the growth and invasive phenotypes of colorectal carcinoma [14], whether LINC01315 could function as a biomarker of TNBC remains unclear.

Mechanism investigations could give a more comprehensive understanding of the significance of LINC01315 in TNBC and benefit the clinical target therapy of TNBC. Sponging microRNAs (miRNAs) is the major pathway of lncRNAs to modulate the development of human cancers. Previously, several miRNAs were identified as the target of LINC01315, through which LINC01315 brought its function into play. In breast cancer, miR-876-5p was negatively correlated with the malignancy of patients and served as a tumor suppressor [15], and it was also revealed to mediate the inhibitory effect of lncRNA FBXL19-AS1 on cell apoptosis of breast cancer [16].

The hypothesis of this study was LINC01315 could mediate the progression of TNBC via regulating miR-876-5p, aiming to provide a potential biomarker and therapeutic target for TNBC.

## Materials and methods

### Tissue samples

Ethics approval has obtained from the Ethics Committee of Yantai Affiliated Hospital of Binzhou Medical University (20,130,019) and written informed consent was obtained from every patient before the enrollment. A total of 103 TNBC patients that received surgical treatment were included from January 2013 to December 2015. Tumor tissues and matched normal tissues were resected during surgery and confirmed by at least two pathologists. Collected tissues were stored in liquid nitrogen at −80°C. The included patients were followed up for 5 years on the telephone to gather their postoperative status information

### Cell culture

Human TNBC cell lines (MDA-MB-231, HCC1806, BT-549, and SUM-159) and normal cell MCF-10 are available from ATCC. Cell culture was performed in the FBS-containing (10%) DMEM culture medium (Invitrogen) at 37°C to the logarithmic period.

### Cell transfection

Small interference RNA of LINC01315 (si-LINC01315, for LINC01315 knockdown, GenePharma) or pcDNA 3.1-LINC01315 (oe-LINC01315, for LINC01315 overexpression) was transfected into TNBC cells. Cell transfection was conducted at room temperature with the help of Lipofectamine 2000 (Invitrogen). Transfected cell cultures are available for the following analyses after 48 h of cell transfection [17].

### Real-time qPCR

Total RNAs of tissue and cell samples were isolated with the Trizol Reagent (Invitrogen). cDNA was synthesized with the PrimeScript RT Reagent Kit (TaKaRa). The expression of LINC001315 and miR-876-5p evaluation was performed with the SYBR Green (Applied Biosystem) and 7900HT Fast Real-Time PCR System (Applied Biosystem). All the data were normalized to GADPH (for LINC01315 and GRK5) or miR-39 (for miR-876-5p) and calculated by the equation 2^−ΔΔCt^. The primer sequences used were as follows: LINC01315 forward 5’-CTGCTGAGCGATGAAGTGGA-3’ and reverse 5’-CTACAGCTGGAGGGAAACCG-3’. miR-876-5p forward 5’-TGAAGTGCTGTGGATTTXTTTGTG-3’ and reverse 5’-TGAATTACTTTGTAAACCACCACCA-3’. GADPH forward 5’-CAGCCTCCAGATCATCAGCA-3’ and reverse 5’-TGTGGTCATGAGTCCTTCCA-3’. miR-39 forward 5’-UCACCGGGUGUAAAUCAGCUUG-3’ and reverse 5’-TCACCGGGTGTAAAT-3’ [13,18,19].

### MTT assay

Cell cultures were seeded at 5 × 10^4^ cells/well into the 96-well plates and starved for 12 h. Cells were supplied with a completed growth medium and incubated for 0, 24, 48, and 72 h. Then, 20 μL MTT solution (Sigma) was mixed with the cultured cells and incubated for another 4 h. The crystal was dissolved by 150 μL DMSO (Sigma) and the absorbance at 490 nm of each well was measured with a Microplate Reader [20,21].

### Transwell assay

Cell cultures were placed in the upper chamber (coated with Matrigel in invasion assay) of the 24-well transwell plates (Corning) and supplied with an FBS-free culture medium. The completed medium was placed in the bottom chamber and incubated at 37°C for 48 h. Then, cells on the upper surface were removed, and the migrated and invaded cells were counted with a microscope (magnification ×200) [20,21].

### Dual-luciferase reporter assay

The binding sites between LINC01315 and miR-876-5p or between miR-876-5p and GRK5 were predicted and cloned into the pmirGLO plasmid to establish the wild type of vectors. While point mutant was conducted on the bindings in the establishment of the mutant type of LINC01315 vector and GRK5 vector. The established vectors were co-transfected with miR-876-5p mimic, miR-876-5p inhibitor, or negative controls into the MDA-MB-231 cell to assess the interaction between LINC01315 and miR-876-5p and between miR-876-5p and GRK5. The luciferase activity of LINC01315 and GRK5 was evaluated with the employment of the Dual-Luciferase Reporter Assay System (Promega) relative to Renilla [20].

### Statistical analysis

The obtained data were presented as mean ±SD of at least 3 independent replicates. Significant differences (*P < 0.05, **P < 0.01, and ***P < 0.001) were estimated with Student’s t-test or one-way ANOVA by GraphPad Prism 7.0. The clinical data were analyzed with the Chi-square test, Kaplan-Meier, and Cox analysis to assess the significance of LINC01315 in TNBC.

## Results

The expression of LINC01315 in TNBC was evaluated in tissue and cell samples, while its role in the disease development and clinical prognosis of patients was assessed. *In vitro*, the effect of LINC01315 on the proliferation, migration, and invasion of TNBC cells was investigated. In mechanism, the involvement of miR-876-5p was verified.

### LINC01315 is upregulated in TNBC

**Figure 1.**
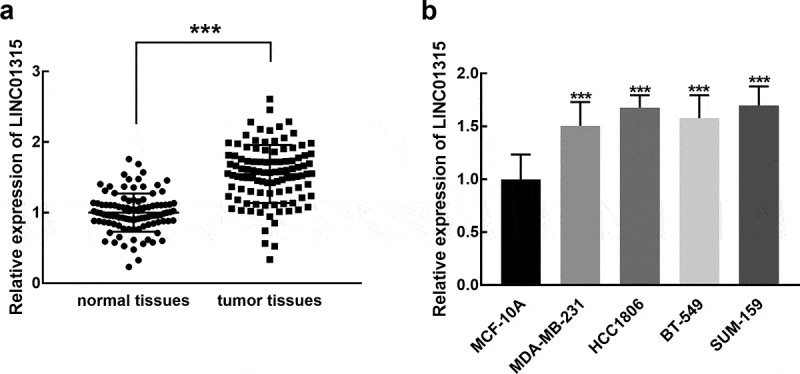
A significant upregulation of LINC01315 was observed in TNBC tissues (**a**) and cells (**b**) compared with normal tissues and cells. Asterisks represent the significant difference. ***P < 0.001.

An elevated expression of LINC01315 was observed in the collected TNBC tissues in comparison with normal tissues, and the difference was statistically significant (*P* < 0.001, Figure 1(a)). Meanwhile, in TNBC cell cultures, including MDA-MB-231, HCC1806, BT-549, and SUM-159 cells, the significant upregulation of LINC01315 was also noted compared with MCF-10A, a normal cell (*P* < 0.001, Figure 1(b)).

### LINC01315 is involved in the development and prognosis of TNBC patients

Based on the average expression of LINC01315 in TNBC tissues, patients were grouped into a low LINC01315 group and a high LINC001315 group. Patients in the high LINC01315 group were found to possess a relatively large tumor size, malignant TNM stage, positive LNM status, and relatively high level of Ki67. Consistently, among the basic clinical features of TNBC patients, the tumor size (*P* = 0.021), TNM stage (*P* = 0.031), LNM status (*P* = 0.007), and Ki67 levels (*P* = 0.025) showed a close association with the expression of LINC01315 (Table 1).

**Table 1.** Association between LINC01315 and clinical features of TNBC patients LNM: lymph node metastasis

		LINC01315		*P* value
	Case (No. 103)	Low (n = 48)	High (n = 55)	
Age (years)				0.626
≤ 50	52	23 (44.23%)	29 (55.77%)	
> 50	51	25 (49.025)	26 (50.98%)	
Tumor size (mm)				0.021
≤ 20	54	31 (57.41%)	23 (42.59%)	
> 20	49	17 (34.69%)	32 (65.31%)	
TNM stage				0.031
I–II	66	36 (54.55%)	30 (45.45%)	
III	37	12 (32.43%)	25 (67.57%)	
LNM				0.007
Absent	54	32 (59.26%)	22 (40.74%)	
present	49	16 (32.65%)	33 (67.35%)	
Menstrual status				0.363
postmenopausal	50	21 (42.00%)	29 (58.00%)	
Others	53	27 (50.94%)	26 (49.06%)	
Ki 67 (%)				0.025
≤ 20	61	34 (55.74%)	27 (44.26%)	
> 20	42	14 (33.33%)	28 (66.67%)	

**Figure 2.**
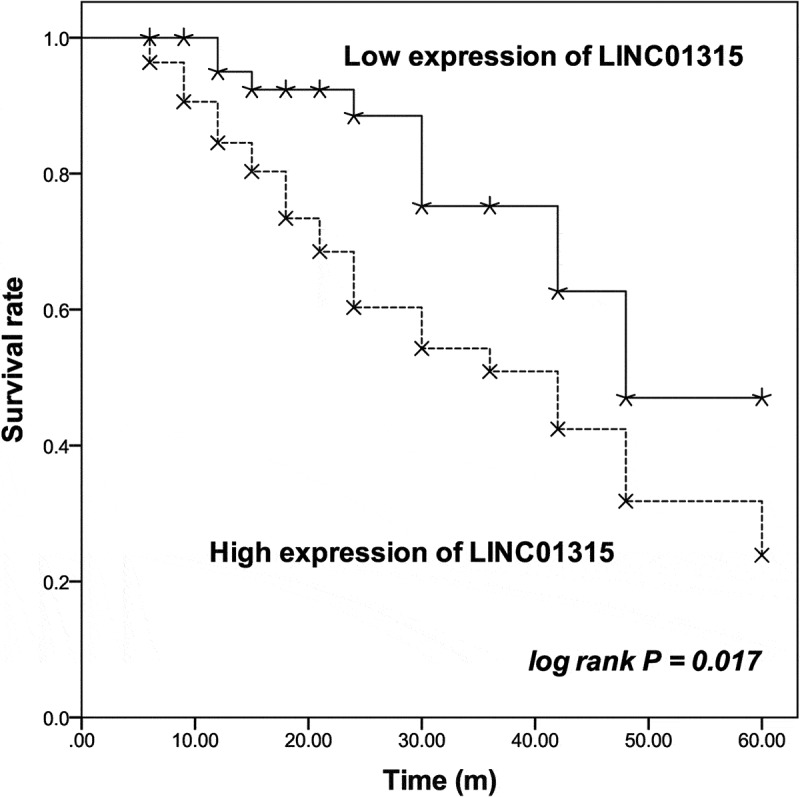
The survival information of patients was plotted by Kaplan-Meier curve followed by log rank test based on the level of LINC01315 in TNBC tissues. The upregulation of LINC01315 was associated with the poor overall survival rate of TNBC patients. solid line: patients in the low expression of LINC01315 group; dash line: patients in the high expression of LINC01315 group. log rank *P* = 0.017.

Additionally, patients in the high LINC001315 group possessed a relatively lower survival rate than patients in the low LINC01315 group (log rank *P* = 0.017, Figure 2). The prognostic value of LINC01315 was also demonstrated by the Cox regression analysis. LINC01315 could independently predict the overall survival of TNBC patients with the HR value of 3.326 (95% CI = 1.426–7.755, *P* = 0.005), as well the TNM stage (HR value = 3.142, 95% CI = 1.178–8.383, *P* = 0.022) and LNM status (HR value = 2.460, 95% CI = 1.145–5.286, *P* = 0.021) of patients (Table 2).

**Table 2.** Multivariate Cox regression analysis evaluating the prognostic value of patients’ clinical features

	HR Value	95% CI	*P* value
LINC01315	3.326	1.426–7.755	0.005
age	1.206	0.614–2.369	0.587
Tumor size	1.381	0.690–2.768	0.362
TNM stage	3.142	1.178–8.383	0.022
LNM	2.460	1.145–5.286	0.021
Menstrual status	1.514	0.742–3.090	0.255
Ki67	1.845	0.876–3.885	0.107

LNM: lymph node metastasis.

### LINC01315 regulates the progression of TNBC cells

**Figure 3.**
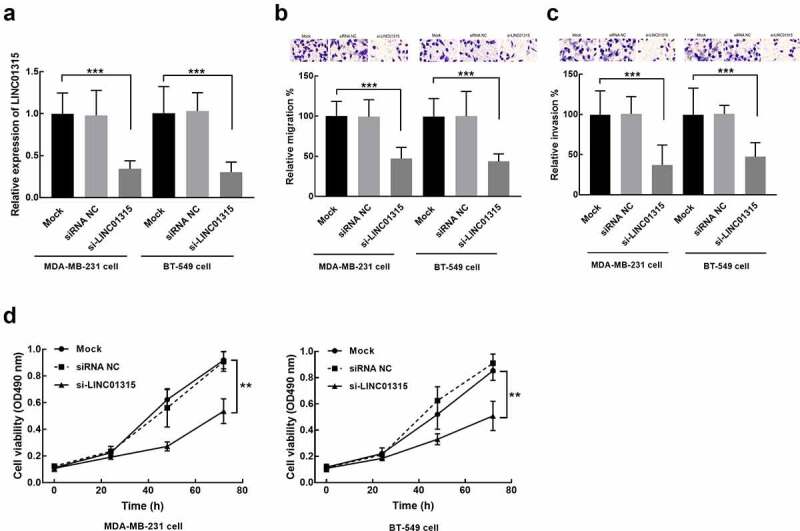
Evaluation of cell transfection efficiency and functional role of LINC01315 in TNBC cells cultured in the DMEM culture medium with 10% FBS. **a**. LINC01315 was suppressed by the transfection of its small interference RNA. **b-d**. The knockdown of LINC01315 dramatically inhibited the migration (b), invasion (c), and proliferation (d) of TNBC cells. Asterisks represent the significant difference. Solid line with circle: mock group (untransfected cells); dash-dotted line: siRNA NC (transfection of siRNA negative control); solid line with triangle: siRNA (transfection of siRNA-LINC01315). **P < 0.01, ***P < 0.001.

MDA-MB-231 and BT-549 cells were selected for *in vitro* experiments due to their relatively high sensitivity to LINC01315 upregulation. The expression of LINC01315 in these two cells was dramatically suppressed by the transfection of its small interference RNA (si-LINC01315, *P* < 0.001, Figure 3(a)).

In MDA-MB-231 and BT-549 cells, the knockdown significantly suppressed cell migration (*P* < 0.001, Figure 3(b)) and invasion (*P* < 0.001, Figure 3(c)). While the consistent inhibitory effect was also observed in the proliferation of these two TNBC cells (*P* < 0.01, Figure 3(d)).

### miR-876-5p and GRK5 mediates the function of LINC01315 in TNBC

**Figure 4.**
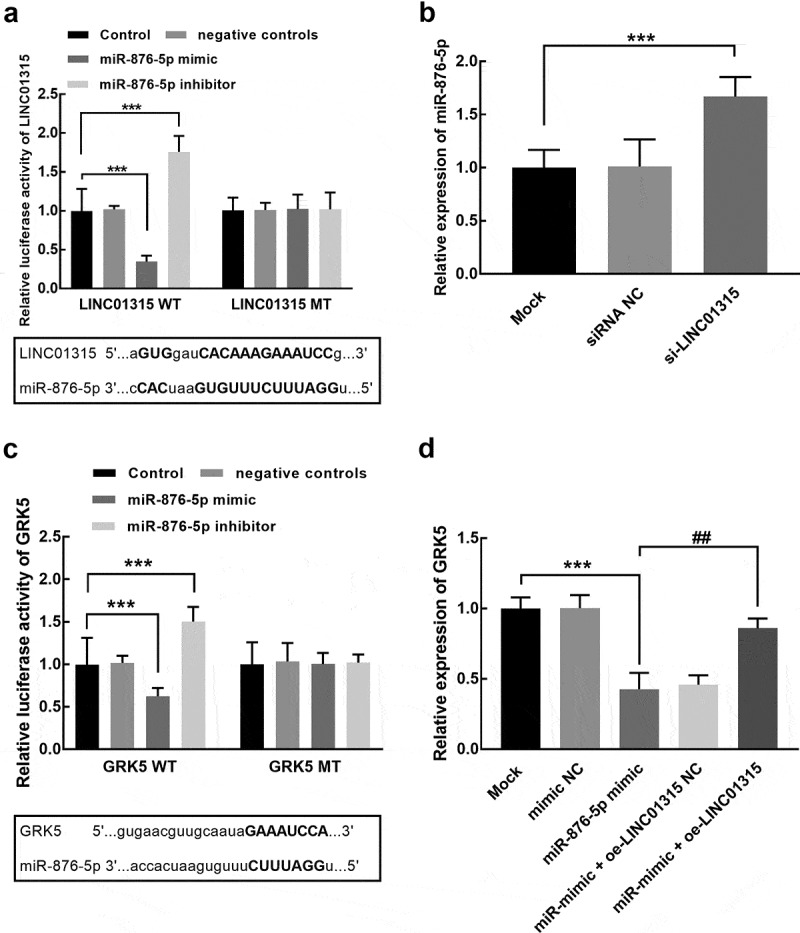
Assessment of the interaction between LINC01315 and miR-876-5p. (**a**). The luciferase activity of the LINC01315 wild-type vector was significantly suppressed by the miR-876-5p overexpression and enhanced by its knockdown. (**b**). The knockdown of LINC01315 significantly promoted the expression of miR-876-5p. Asterisks represent the significant difference. (**c**). The luciferase activity of GRK5 was dramatically suppressed by the overexpression of miR-876-5p and enhanced by its knockdown. (**d**). The elevation of miR-876-5p suppressed the expression of GRK5, which was reversed by LINC01315 overexpression. ***P < 0.001 compared with the mock group. ^##^*P* < 0.01 compared with the miR-876-5p group.

The overexpression of miR-876-5p led to a significant inhibition in the luciferase activity of LINC01315, which was significantly enhanced by miR-876-5p silencing (*P* < 0.001, Figure 4(a)). On the other hand, the expression of miR-876-5p was also promoted by the knockdown of LINC01315 (*P* < 0.001, Figure 4(b)). For the downstream target of miR-876-5p, GRK5 was predicted as its direct target. The overexpression of miR-876-5p was found to inhibit the luciferase activity of GRK5, which was promoted by miR-876-5p knockdown (*P* < 0.001, Figure 4(c)). The expression level of GRK5 was also dramatically suppressed by miR-876-5p overexpression, which was reversed by the elevation of LINC01315 (*P* < 0.01, *P* < 0.001, Figure 4(d)).

## Discussion

Improving the survival rate and monitoring the development of TNBC remains a challenge for women’s health worldwide [22,23]. A number of studies have indicated the involvement and the biomarker role of lncRNAs, especially differently expression lncRNAs, in TNBC progression and prognosis [24,25]. For instance, lncRNA H19 promoted the metastasis and invasion of TNBC cells via regulating the p53/TNFAIP8 pathway [26]. Downregulated lncRNA NEF could differentiate TNBC patients from healthy controls and predict the poor prognosis of patients [27]. Here, the upregulation of LINC01315 was disclosed in TNBC, which was positively correlated with the crucial clinicopathological characteristic of TNBC patients, such as TNM stage, LNM status, and tumor size. The LINC01315-related features of patients are believed to represent the disease progression and severity of patients, suggesting the involvement of LINC01315 in TNBC progression [28]. Additionally, TNBC patients’ overall survival also showed a negative correlation with the expression of LINC001315, indicating that LINC01315 could function as a prognostic biomarker of TNBC.

In previous studies, the abnormal expression of LINC01315 was controversial in different cancers. Meanwhile, dysregulated LINC01315 was also reported to play roles in the development of various malignant tumors. In oral squamous cell carcinoma (OSCC), LINC01315 was downregulated and showed a dramatically inhibitory effect on the proliferation, migration, and invasion of OSCC cells, and dampened cell apoptosis [12]. Otherwise, a significant upregulation of LINC01315 was observed in colorectal carcinoma accompanied by the enhancement of the tumorigenicity and sponging of miR-205-3p [14]. Similarly, in papillary thyroid cancer, the expression level of LIN01315 was significantly higher than noncancerous tissues or normal cells, which was found to accelerate cell colony formation and invasion via regulating miR-497-5p [13]. The upregulation of LINC01315 in this study was reversed by cell transfection of LINC01315 interference RNA, which significantly inhibited the main cellular processes of TNBC and was consistent with its function in colorectal carcinoma and papillary thyroid cancer, indicating the tumor enhancer role of LINC01315 in TNBC.

Uncovering the mechanism of lncRNA regulatory effect on tumor progression would be beneficial for developing a novel therapeutic strategy for TNBC. It has been widely known that lncRNA could regulate miRNAs to display its enhanced or inhibitory effect on tumor progression [29]. For example, miR-150 served as the direct target of lncRNA CASC11 during the promoted effect of lncRNA CASC11 on prostate cancer [30]. Previous studies mentioned several miRNAs that mediate the regulatory effect of LINC01315 in various cancer, such as miR-211, miR-205-3p, and miR-497-5p [12–14]. miR-876-5p was predicted to be sponged by LINC01315 and their interaction was validated in the present study. miR-876-5p was revealed to be a tumor promoter in breast cancer and was also involved in the regulatory effect of lncRNA FBXL19-AS1 [15,16]. Furthermore, the downstream target of miR-876-5p, GRK5, was disclosed. GRK5 was reported to serve as a tumor enhancer of breast cancer, of which the downregulation hampers the tumor progression [31]. Therefore, LINC01315 was speculated to regulate the progression of TNBC through the miR-876-5p/GRK5 axis.

However, the deep molecular mechanism was not dug out by the present results. Although the interaction between LINC01315 and miR-876-5p has been validated in the present study, the direct evidence of miR-876-5p mediates the regulation of TNBC progression by LINC01315 lacked. Therefore, further investigations are needed to evaluate the specific function of miR-876-5p during the tumor promoter role of LINC01315. On the other hand, the size of clinical data was not large enough to obtain typical results. For instance, except for TNM stage and LNM status, tumor size and Ki67 level of patients could also predict the prognosis of TNBC patients to some degree [32–34]. But the prognostic implication of these characteristics of patients was not obvious in the present study, which might result from the limited sample size [35,36]. Hence, a larger sample size may be a crucial factor in future studies.

## Conclusion

Taken together, LINC01315 was identified as an upregulated lncRNA in TNBC, which was involved in disease development and indicated patients’ poor prognosis. Furthermore, LINC01315 was also a tumor promoter of TNBC that modulated the progression of TNBC via modulating miR-876-5p/GRK5.

## Supplementary Material

Supplemental Material
