# Supplementary figures and images for: lncRNA LINC01315 promotes malignancy of triple-negative breast cancer and predicts poor outcomes by modulating microRNA-876-5p/GRK5
Source: Bioengineered. 2022 Apr 12;13(4):10001–9. doi: 10.1080/21655979.2022.2062536 (PMC9161853; doi:10.1080/21655979.2022.2062536)

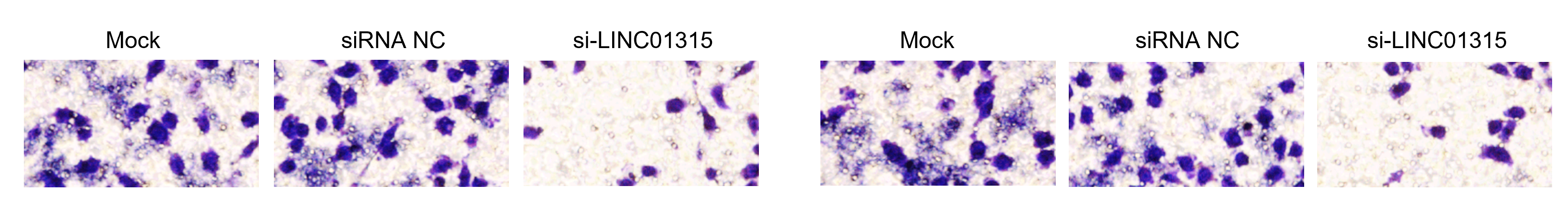

Supplement: Supplemental Material [file KBIE_A_2062536_SM2111.zip › Supplementary material invasion.tif]

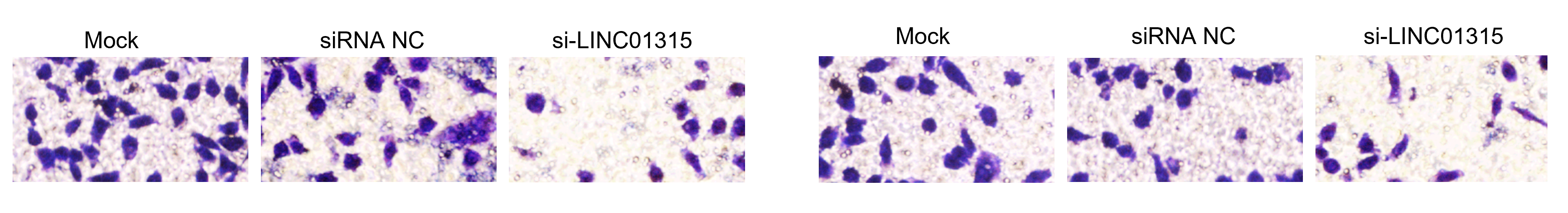

Supplement: Supplemental Material [file KBIE_A_2062536_SM2111.zip › Supplementary Matrial migration.tif]
